# Climbing Technique Evaluation by Means of Skeleton Video Stream Analysis

**DOI:** 10.3390/s23198216

**Published:** 2023-10-01

**Authors:** Raul Beltrán Beltrán, Julia Richter, Guido Köstermeyer, Ulrich Heinkel

**Affiliations:** 1 Professorship Circuit and System Design, Chemnitz University of Technology, Reichenhainer Straße 70, 09126 Chemnitz, Germany; j.richter@jrarts.de (J.R.); heinkel@hrz.tu-chemnitz.de (U.H.); 2 Department Sportwissenschaft und Sport, Friedrich-Alexander-Universität Erlangen-Nürnberg, Schlossplatz 4, 91054 Erlangen, Germany; guido.koestermeyer@fau.de

**Keywords:** climbing motion analysis, sports and computer science, video analysis, key point detection, human pose estimation

## Abstract

Due to the growing interest in climbing, increasing importance has been given to research in the field of non-invasive, camera-based motion analysis. While existing work uses invasive technologies such as wearables or modified walls and holds, or focuses on competitive sports, we for the first time present a system that uses video analysis to automatically recognize six movement errors that are typical for novices with limited climbing experience. Climbing a complete route consists of three repetitive climbing phases. Therefore, a characteristic joint arrangement may be detected as an error in a specific climbing phase, while this exact arrangement may not considered to be an error in another climbing phase. That is why we introduced a finite state machine to determine the current phase and to check for errors that commonly occur in the current phase. The transition between the phases depends on which joints are being used. To capture joint movements, we use a fourth-generation iPad Pro with LiDAR to record climbing sequences in which we convert the climber’s 2-D skeleton provided by the Vision framework from Apple into 3-D joints using the LiDAR depth information. Thereupon, we introduced a method that derives whether a joint moves or not, determining the current phase. Finally, the 3-D joints are analyzed with respect to defined characteristic joint arrangements to identify possible motion errors. To present the feedback to the climber, we imitate a virtual mentor by realizing an application on the iPad that creates an analysis immediately after the climber has finished the route by pointing out the detected errors and by giving suggestions for improvement. Quantitative tests with three experienced climbers that were able to climb reference routes without any errors and intentionally with errors resulted in precision–recall curves evaluating the error detection performance. The results demonstrate that while the number of false positives is still in an acceptable range, the number of detected errors is sufficient to provide climbing novices with adequate suggestions for improvement. Moreover, our study reveals limitations that mainly originate from incorrect joint localizations caused by the LiDAR sensor range. With human pose estimation becoming increasingly reliable and with the advance of sensor capabilities, these limitations will have a decreasing impact on our system performance.

## 1. Introduction

Climbing as a sport has become increasingly popular, and its practice has spread to the point that today we can find climbing walls not only in specialized gyms, but also in public parks, with attractive designs for children and beginners who are encouraged to take up this sporting trend. Bouldering is a variant of climbing without the use of ropes, offering its practitioners different climbing challenges that they can perform without the help of a partner and individually. Unlike other solo sports such as cycling or running, bouldering does not have many tools for the practitioner to control and measure their exercise autonomously [1], hence there is a need for applications to measure, analyze, and provide feedback to people in this discipline. Additionally, the proliferation of wearables to capture information about the activity of the human body has aroused special interest in athletes and trainers who see the need for additional tools that allow them to collect data with the aim of analyzing their routines and improving their performance.

Climbing requires the development of some physical skills that differentiate it from other sports. Among them is that it requires efficient movements in order to make proper use of body energy to reach the target on a route, either lateral or ascending [2]. It also requires the development of strength in small muscles, psychological stress management in the face of a potential fall, and a visual–motor ability to visualize and reach different holds on a random route when the route has not been predefined [3]. The different movements performed by a climber are framed within one of three clearly defined stages, referred to here as *phases*, which can be analyzed in relation to the climber’s pose and the speed of the body joints involved in the action [4].

Within the climbing phases, as well as in the transition between them, it is common for the climber to make mistakes in posture and hip movement, especially when they are a beginner. These mistakes are part of learning and the continuous improvement in climbing technique, and their early correction prepares the climber for different climbing situations where maximum strength and resistance will be required. The climbing errors are usually corrected with the assistance of a more experienced partner who acts as a guide, pointing out the error and demonstrating the correct execution of the exercise.

To determine the current phase and check whether the climber makes typical errors while in it, we introduce a finite state machine in which the transition between states depends on which joints are in motion. The joint movement is detected from RGB-D video recordings made with an iPad Pro 4th Generation, which has a LiDAR sensor and provides us with Vision [5], Apple Inc.’s framework for human pose estimation (HPE). The Vision information is complemented with the LiDAR data to obtain a 3-D model of the climber’s pose to establish whether the joint is in motion by means of an algorithm. Simultaneously, we determine the angles and relationships between joints that will allow us to evaluate the existence of climbing errors.

In this study, we present a novel tool that acts as a virtual trainer, allowing the video recording of the climber and pointing out errors, as well as providing feedback to correct them. In the development of this tool, we modeled six of the frequent errors in bouldering and analyzed, among other variables, the position and velocities of the subject’s hands, feet, and hips. Additionally, we propose a model of transitions between climbing phases based on the position of the climber’s limbs and their center of mass (CoM).

The paper is organized in five sections. Following the introduction, in Section 2, we include a summary of similar related work for HPE in the climbing domain, using non-invasive sensors and climbing analysis. In Section 3, theoretical concepts on climbing phases and errors are presented. Section 4 explains our modeling of the different cases and artifacts needed for our application. In Section 5, we carry out the evaluation, first presenting the methodology and then analyzing the results. Finally, Section 6 draws conclusions and provides proposals for future work.

## 2. Related Work

To provide unified information on existing bouldering research from different perspectives like sensors, HPE, and motion analysis algorithms, ref. [6] presents a survey including existing studies using optical devices, wearables, and capacitive force sensors. There, they list commercial and open source HPE frameworks, highlighting the difficulties in sport climbing when faced with occluded limbs and the climber’s pose taken from the back. The study also points out the challenge of tool development for teaching and training sport climbing. Likewise, an interesting classification of sensors used in climbing, both indoors and outdoors, is included in [3]. Several groups are presented there that allow comparison in terms of invasive and non-invasive sensors and practical benefits and limitations, in addition to performance metrics. More specifically, we find studies that use a particular type of sensor to measure parameters such as force, position, and velocity. These sensors may be part of instrumented climbing holds [7,8,9,10,11,12], attached to the climber’s body [13,14,15], or attached as visual markers [16,17,18,19]. Some researchers also highlight the benefits of non-invasive sensors, such as cameras, where measurements are made without contact and without affecting climbers, walls, or holds [20,21].

An important topic for climbing analysis is the HPE research area. Ref. [22] presents a comprehensive survey of the most relevant publications since 2014, describing techniques based on deep learning and datasets for 2-D and 3-D, silhouette, and skeleton extraction. The survey summarizes the challenges for the algorithms, such as occlusions and depth data ambiguity errors, in addition to the lack of sufficient training data for certain scenarios. In this regard, ref. [23] extends the information by including specific sports and physical exercises, focusing the study on markerless and camera-based systems. They point out numerous publications that combine general-purpose HPE techniques for 2-D skeletal prediction and their subsequent pairing with depth data to build 3-D models, a solution to tackle the lack of datasets for a given sports scenario.

In research on climbing motion analysis, ref. [18] made early studies to compare entropy, force, and speed by using markers attached to the climbers and tracking them through a single camera. More recently, ref. [16] used a similar concept of markers and a video capture system to compare the distance of climbers to the wall using the CoM. In the field of speed climbing, ref. [19] measured the energy performance of climbers when making horizontal advances on the climbing route, i.e., when executing moves to lateral positions. They calculated the 3-D trajectory and measured the climber’s speed using two drones armed with cameras following a marker attached to the climber. Similarly, a novel method for analyzing the climber’s velocity from non-static video sequences is presented in [20]. The authors avoid invasive techniques by using only a moving camera to measure the position, velocity, and acceleration of the CoM. They define a set of relevant body joints to calculate the angles of the body parts and the time taken to reach adjacent holds. These parameters allow different climbers to be evaluated on the same wall configuration, providing students and athletes with comparative results on speed, movement, and location on the climbing route. To follow the climber along the speed wall, the camera position and distance to the wall are algorithmically determined, and image processing is used for segmentation, feature detection, and matching. Especially, an analysis of the change in knee and elbow angles along the route execution is provided, so that trainers can detect problems in the climbing technique by comparing it with the same execution of other expert athletes. In climbing, so-called smart materials, such as capacitive sensors that can measure the presence of a climber [12], can also be employed for analyzing climbing movements. An insight into the latest developments in smart materials is provided by [24].

In application development for sport climbing, ref. [15] presented a bouldering assistance system that projects a reference shadow on the climbing wall to guide the climber in the movements to follow. This assistant, called betaCube [25], locates the subject using a 3D camera and allows them to follow the projection of climbing sequences pre-recorded with the same system, applying an augmented reality concept. More recently, ref. [1] presented a tool that provides the climber with an analysis of their climbing from video sequences and the use of machine learning (ML). The project included video sequences of climbers with different experience in various scenarios. The tool produces an automatic output with information on the percentage of the route completed, the number of moves made, and the identification of route parts to be improved based on a proposed algorithm that uses the climber’s pose and the time spent on each hold. The segmentation of the climbing holds is achieved using predictive models based on YOLO [26] by means of Roboflow [27]. The climber’s pose is estimated using MediaPipe [28], an ML framework capable of inferring 3-D landmarks and segmenting the climber. In the same climbing motion analysis scenario, a video recording system is proposed in [29] to automatically detect movement errors common to novice climbers. The system acts as a virtual mentor providing graphical feedback with a rich user interface developed using Apple Inc.’s ARKit [30].

## 3. Background

In this section, we briefly introduce concepts necessary to frame our work: the division of the climbing stages for their study; the most common errors that we identified within these stages and that are the object of our analysis; and finally, we describe a mathematical method used for video synchronization.

### 3.1. Climbing Phases

According to [31], there are three stages into which a climber’s actions can be split:

- Preparation, when the climber sets up their body by establishing the correct position and setting their feet to initiate a standing-up action.
- Reaching, when the climber is in the action of standing up to reach and grab the next hold on an ascending climbing route.
- Stabilization, when the climber adjusts and relaxes the body after having reached the hold before starting the next series of movements.

**Figure 1:**
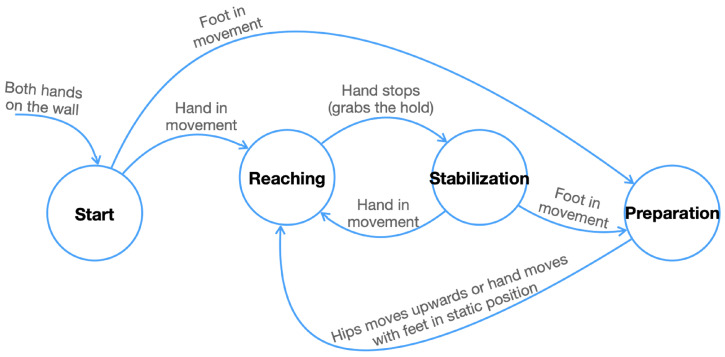
Proposed climbing phases transition state diagram.

In these stages, the climber performs different arm, leg, and hip movements sequentially and at specific times. For example, the climber first reaches for a hold with one hand, stabilizes the body in an attempt to conserve energy, and then places the feet; finally, the climber rises to grab the next hold with the other hand. These three divisions of the climber’s actions are what we have referred to here as phases. Figure 1 presents the transition diagram for the climbing phases, which will be described in terms of joint movements in Section 4.6.

### 3.2. Climbing Errors

A correct climbing technique aims at optimizing the climber’s effort to reach the holds in the execution of a climbing route, in addition to preventing possible injuries. Six basic climbing techniques are presented below to verify the correct execution of the climbing action, including the characteristic errors related to the climber’s position or limb movements. Constant values for the elbow and shoulder angles, the time of the reaching action, and the hip distance difference when comparing climbers were given as a reference by our sport climbing expert, but some of these were updated later in the tuning of the algorithms.

#### 3.2.1. Decoupling

This is an energy-saving technique in the preparation phase, where the arm of the holding hand has to be straight when the feet are being set. Here, the holding hand is defined as the hand that is higher up and holds the main weight of the body, while the hand in the lower position is named the supporting hand and holds the body in the direction of the wall.

Characteristic error: The elbow angle and the shoulder angle both are less than 150°.

#### 3.2.2. Reaching Hand Supports

In the reaching phase, the supporting hand should stay as long as possible on the hold before reaching to the next hold and becoming the new holding hand.

Characteristic error: Reaching takes longer than 1 s.

#### 3.2.3. Weight Shift

In the reaching phase, the weight should be shifted onto the leg that is opposite the supporting hand: the knee is shifted vertically in front of the toe of this leg, the weight of the body, or the hips, is shifted first over the leg towards the wall and then upwards.

Characteristic error: The climber stands while pulling with the holding arm, and in the course of the movement the knee is never vertical in front of the toe.

#### 3.2.4. Both Feet Set

In this movement, both feet should be placed onto the wall in the standing up action during the reaching phase. One foot can also be simply pressed against the wall; it does not necessarily have to be on a bolted step.

Characteristic error: Only one foot has wall contact when standing up, while the other foot continues to move or hangs loosely in the air.

#### 3.2.5. Shoulder Relaxing

In the stabilization phase, after gripping, the arm of the new holding hand should be stretched again and the CoM should be lowered again, although it may be higher. The weight of the body, or the hips, approaches the perpendicular of the holding hand again, and the distance depends on what the climber can do with the second hand.

Characteristic error: After gripping, the arm of the new holding hand remains locked and the angle of the elbow and shoulder does not open, and the inner angle of the elbow and the angles of the shoulder in the dorsal and sagittal plane are less than 150°.

#### 3.2.6. Hip Close to the Wall

The aim is to keep the hips as close as possible to the wall within the reaching phase. This mobility of the hips results in more efficient and economical climbing, as most of the body weight rests on the toe holds.

Characteristic error: The assessment is made by reference to another climber who performs the same route in precise form. When comparing the distance to the wall of the climber’s hips to the reference, the distance should not be exceeded by more than 5 cm. Climbers of similar size to the reference are considered here.

### 3.3. Dynamic Time Warping

Especially for hip-close-to-the-wall error detection, in our approach, dynamic time warping (DTW) is used to align the video sequence of an expert climber with the novice sequence. DTW is a measure of similarity between two time sequences, also referred to as curves, represented as discrete sets in a common metric space [32]. These sequences may vary in speed, and DTW allows us to correlate all the points of one set into the other, making a one-to-many match that covers all the valleys and peaks of both curves. Having $A=(p_{1},\ldots ,p_{n})$ and $B=(q_{1},\ldots ,q_{m})$, to create an ordered *coupling* $C=(c_{1},\ldots ,c_{k})$, the algorithm satisfies the following rules:

- All points of the two sequences must match in both directions with the heads and tails paired, such that $c_{1}=\left(p_{1},q_{1}\right),c_{k}=\left(p_{n},q_{m}\right)$.
- The indices mapping the first sequence to the other must be monotonically increasing and vice versa, $c_{r}=\left(p_{i},q_{j}\right)\Rightarrow c_{r+1}\in \left\{\left(p_{i+1},q_{j}\right),\left(p_{i},q_{j+1}\right),\left(p_{i+1},q_{j+1}\right)\right\}$, for r<k, i.e., cross-matching is not allowed.

Hence, as [32] describes, the DTW distance between A and B is given by Equation (1).

(1) $$dtw\left(A,B\right)=\min_{C:coupling}\sum_{\begin{array}{c} (p_{i},q_{j})\in C \end{array}}dist\left(p_{i},q_{j}\right)$$

This technique can be used to compare two time series with different lengths and speeds, to distinguish the underlying pattern rather than looking for an exact match in the unprocessed sequences. As Equation (1) shows, the usual Euclidean distance between the two signals is replaced by a dynamically adjusted metric dist that allows the aligning, preserving the temporal dynamics of the sequences by directly modeling the correspondence of the two time series at each point.

## 4. Methods and Implementation

In the following we describe in detail our proposed model for each of the climbing analysis components. These are estimation of the climber’s pose by estimating the skeletal joints, segmentation of the climbing wall and establishment of the coordinate system, identification of the beginning and end of the climbing route in the video, alignment of the reference and climber videos, segmentation of the climber’s movements, the transition rules between the climbing phases, and finally, the error detection metrics.

### 4.1. Climber Pose

As noted by [23], although many public general-purpose HPE systems exist, none of them are trained in specific fields of sport and physical exercise. In addition to this, there are situations such as occlusions, that affect the detection accuracy of the climber’s skeleton. Nevertheless, for the assessment of our climbing algorithms we have obtained acceptable results using Vision [5], the HPE framework provided by Apple Inc. In particular, we have chosen an iPad Pro 4th Generation equipped with a LiDAR sensor, whose integration of hardware and software in a single device is practical for climbing applications.

**Figure 2:**
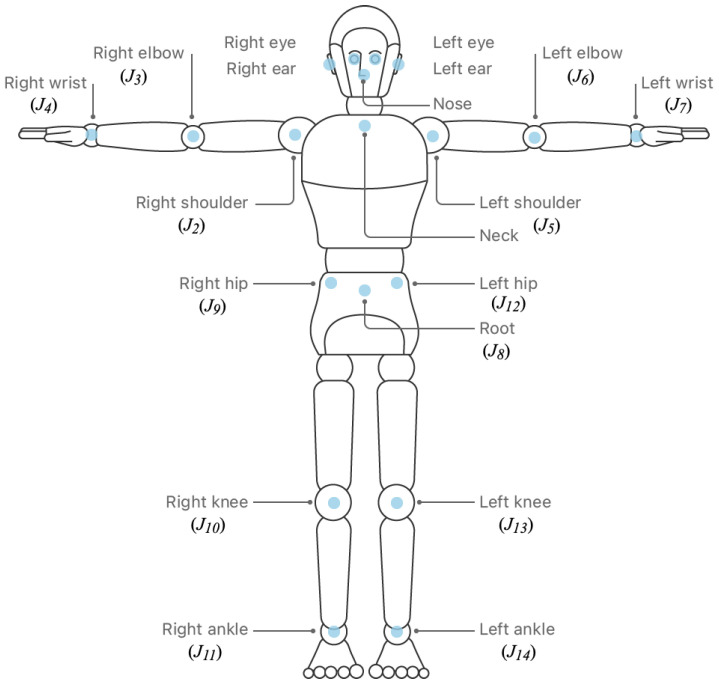
Relevant body joints in climbing analysis. Image source [5].

**Figure 3:**
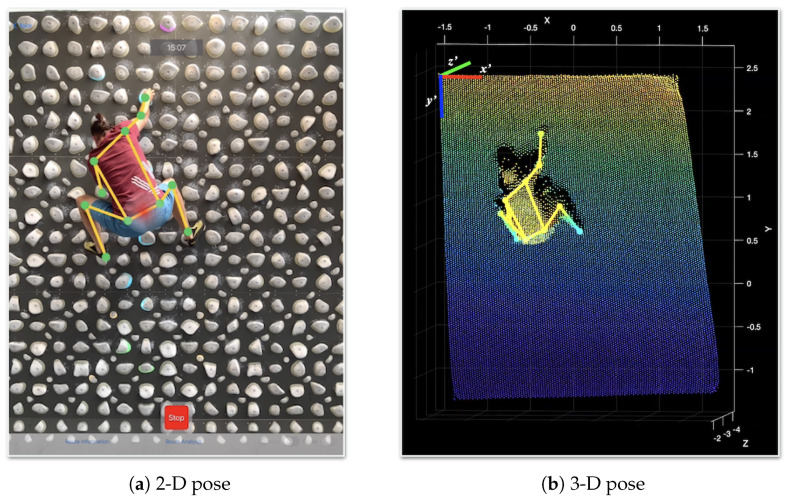
Extraction of the climber’s pose in a video frame. (**a**) Skeleton provided by Vision and calculation of the climber’s CoM, the latter highlighted by a red point. (**b**) Skeleton projection on the point cloud to assign the depth component to each skeleton joint.

Vision works in the 2-D space, processing RGB video frames at a maximum sample rate of 60 frames per second (FPS), thus capturing 60 poses per second in our implementation. Each pose consists in turn of 19 body joints given in Cartesian camera coordinates ($R_{x},R_{y}$), of which we have taken 13 for our evaluations, as shown in Figure 2. Each of these coordinates is mapped to the depth data acquired by the LiDAR sensor to obtain the depth component $R_{z}$, as shown in Figure 3; thus, building the 3-D skeleton for each frame, expressed as the set *J* in Equation (2).

(2) $$J=\{J_{k}={\left(R_{x},R_{y},R_{z}\right)}_{k}:J\in R^{3}\;,\;2⩽k⩽14\}$$

Considering that the device memory is scarce when processing several video frames in parallel, as explained later in Section 4.8, the depth information does not exist for each pixel of the image and instead is obtained by means of a depth grid that associates each point with reticular image sections. Hence, the 2-D coordinates of the joint, obtained with Vision, fall within one of the grid squares and must be associated with the nearest point to determine the third coordinate. This is achieved by applying a kd-tree algorithm [33] on the nine grid points in the joint vicinity and averaging the depth measurements of the three closest points, thus obtaining the joint’s depth measurement.

### 4.2. Wall Plane Model

The 3-D skeleton is initially built on the reference coordinate system provided by the camera, named the camera system. To carry out the position calculations with the skeleton joints independently of the camera system, it is necessary to transfer them to their own reference coordinate system, which is constructed from the climbing wall. Therefore, we call this new coordinate system, the wall coordinate system. This process is performed in a first extrinsic calibration step, detailed below.

In our study, the climbing wall is represented as a rectangular plane with an optional tilt. This plane and its boundaries are determined from the analysis of the point cloud from a random frame in the first 3 s of the video, where only the climbing wall is in the scene. The plane equation is determined using RANSAC [34], and the point cloud of the climbing wall is segmented to determine the edges of the rectangle by a 2-D polygonal approximation [35].

We choose the upper left corner of the wall rectangle as the origin of the new coordinate system and construct the transformation matrix *T* as shown in Equation (3). The rotation matrix *R* in Equation (4) results from Rodrigues’ rotation formula, by including the wall plane normal vector *n* and the unit vector in the *z*-direction of the camera model, and subtracting the identity matrix *I* [36]. Lastly, the transformation matrix uses a translation vector *t* from the camera origin to the wall coordinate origin. All skeleton joints $J_{k}$ are rotated and translated by applying the transformation shown in Equation (5), hence the distances calculated in independent videos will be relative to the same coordinate system located on the wall. This procedure allows us to compare different recordings with different orientations of the camera with respect to the wall.

(3) $$R=2\cdot \frac{\left(z+n\right){\left(z+n\right)}^{⊺}}{{\left(z+n\right)}^{⊺}\left(z+n\right)}-I$$

(4) $$T=\left[\begin{array}{cc} R & t\\ 0 & 1 \end{array}\right]$$

(5) $$J_{k,wall}=T\times J_{k,camera}$$

### 4.3. Automatic Route Delimitation

In bouldering, it is natural for a climber to ascend the climbing wall and then, at the end, jump, or descend by *un-climbing* their steps. Determining the start and end points of a climbing route automatically is an important task that allows us to differentiate the portion of the video that is subject to comparison and analysis. Technically, two moments are evaluated, namely:

(i) In the first half of the video, we look for the frame in which the hip position is the lowest to the origin of the wall coordinates to obtain the start of the portion.
(ii) In the second part of the video, we take the last frame in which one of the two hands registered the highest position to obtain the end of the portion.

Hence, the starting point $r_{s}$ is taken as the first frame where the *y*-position of the hip $J_{8}$ is the lowest, beginning the route; and the end point $r_{e}$ is taken as the last frame where the *y*-position of either wrist, $J_{7}$ or $J_{4}$, reaches the highest point along the climbing route, as specified in Equations (6) and (7).

(6) $$\;r_{s}=min\left(f^{*}\right):J_{8y,f^{*}}⩽J_{8y,f_{i}}\;\forall \;f_{i}\in \left[0,\#F/2\right)$$

(7) $$r_{e}=max\left(f^{*}\right):J_{7y,f^{*}}⩾J_{7y,f_{i}}\;\vee \;J_{4y,f^{*}}⩾J_{4y,f_{i}}\forall f_{i}\in \left[\#F/2,\#F\right)$$

### 4.4. DTW Alignment

Having a reference climbing route recorded by an expert in a video sequence $C^{\prime }$, a novice climber repeats the same route, giving a second video recording $C^{\prime \prime }$. For the simultaneous analysis of these two recordings, we use the DTW technique applied to the trajectory of the climber’s hips $J_{8}$ in both videos. The trajectory of $J_{8}$ is projected into the *x*–*y* space of the climbing wall model in each case. The Euclidean distance between the projected joint and the wall origin is then calculated as the distance metric dist, thus forming two independent time series of length equal to the number of frames in each video.

**Figure 4:**
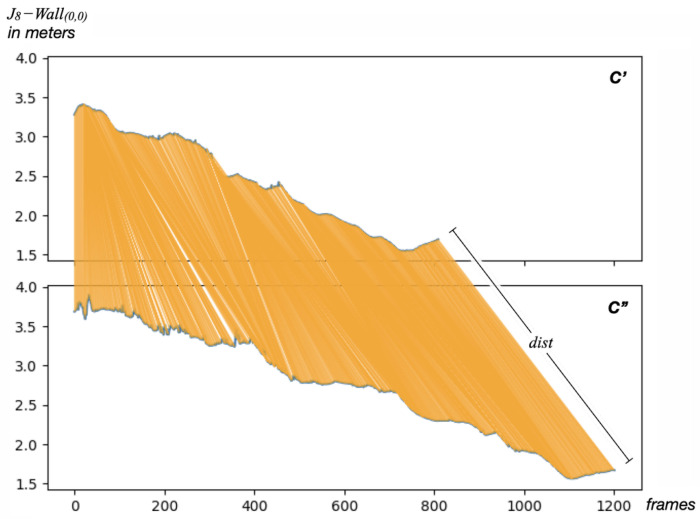
Correlation of two different recordings $C^{\prime }$ and $C^{\prime \prime }$ on the same climbing route.

As shown in Figure 4, after applying DTW, each frame of $C^{\prime }$ corresponds to a frame of $C^{\prime \prime }$. This relationship, not necessarily simultaneous or unique, can be represented as a series of tuples relating the position of the hips of the climber in each frame of both videos; hence, we can define $W_{C}$ as the alignment of the two videos, as shown in Equation (8).

(8) $$W_{C}=\left\{\left(f_{i},f_{j}\right)\;|\;dtw{\left(C^{\prime },C^{\prime \prime }\right)}_{J_{8}}\;,\;\forall f_{i}\in C^{\prime }\;,\;\forall f_{j}\in C^{\prime \prime }\right\}$$

### 4.5. Motion Segmentation

The transition between the phases is given by the movements of hands, feet, and the hips, hence identifying the range of frames in which the joints are in motion or static is a preliminary step to determine the phase in which the climber is. The segmentation of the video frames in which these joints are in motion is performed with a projection of the skeleton in the *x*–*y* plane of the wall coordinate system. There, the velocity of the joints is evaluated independently, and the ranges of motion are established for each joint.

In [21], a motion segmentation algorithm is proposed for each of the climber’s body joints that are of interest in the analysis of climbing. The technique used is based on a *standard score* [37] on the joint velocity signal sampled at each frame of an RGB video recording. In this procedure, the mean of the velocity signal is taken and the standard score, or z-score, is calculated as the number of standard deviations the velocity is above or below the mean in each frame, according to Equation (9). The new z-score signal allows the tracking of prominent changes in the velocity’s mean, related to joint movement intervals. The original method looks for crossover points between the nth-standard-deviation (*n*-σ) graph and the velocity signal, thus marking the initial and final frames of a probable joint motion. The presented algorithm detects the motion of each key joint independently in a given sequence of climbing poses, and shows good results when the signal has well-defined velocity peaks, but is prone to errors when the joint signal is affected by noise. This noise is mainly introduced by jittering in the detection of the joint position, which translates as joint motion, and this in turn into false positives in the algorithm results.

(9) $$z=\frac{v-\mu }{\sigma }$$

**Figure 5:**
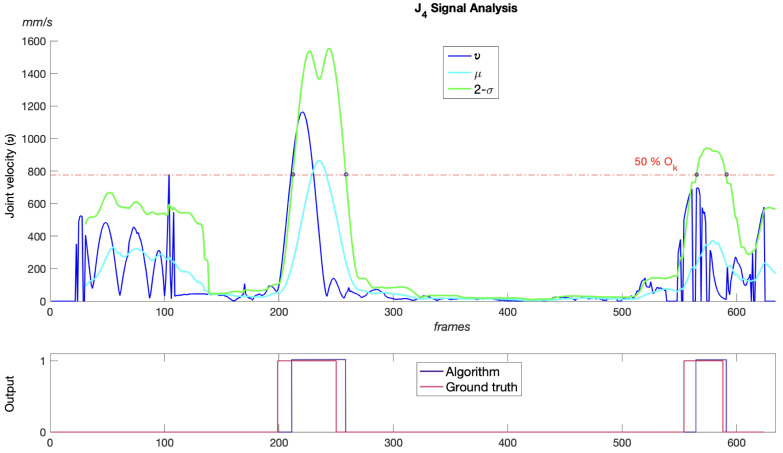
nth-standard-deviation graph with threshold of 50% of its maximum peak $O_{k}$. Here, n=2 and $O_{k}=1580$ mm/s.

Making use of the same standard score technique as described above, we present here a variation in the selection of crossover points to determine the initial and final frames that define the motion segment of the key joint, which is described as follows. By analyzing *n*-σ, it is possible to use only this single graph to extract the points where the signal gradient increases and decreases rapidly by comparing the *n*-σ against a 50% threshold of its maximum $O_{k}$. This threshold results after observing that *n*-σ sharpens non-jittering velocity increments so that we can extract such increments by finding the intersection of the graph with its complement, $O_{k}-n$-σ, which coincide in the middle of the graph. This technique makes it possible to skip those short velocity peaks introduced by jittering in the skeleton detection, as can be seen for the first 100 frames in the example in Figure 5.

The result of the algorithm is a set of intervals for each of the six key joints $J_{7},J_{4},J_{8},J_{14}$, and $J_{11}$. A joint $J_{k}$ is then considered to be in motion for a given frame $f_{i}$ if the frame belongs to one of the motion segmentation intervals $J_{M}$, as indicated in Equation (10). With *m* and *n* as the limits of the intervals, $K_{\mu }$ as the minimum number of frames for a motion to be considered valid, and *l* as the number of detected movements. For validation of whether the joint is static, the complement $\bar{J_{M}}$ is used.

(10) $$J_{M_{k}}=\left\{f_{i}\in {\left[f_{m},f_{n}\right]}_{l}\;|\;m⩽i⩽n\;,\;K_{\mu }⩽n-m\;,\;l\in N\right\}$$

### 4.6. Phase Transitions

The climbing analysis is performed by dividing the video sequence *C* into the climbing phases: preparation, reaching, and stabilization. To move from one phase to the next, the climber’s key joints must fulfill specific criteria, which are described below.

#### 4.6.1. Preparation

From stabilization, we transfer to the preparation phase if the feet start moving. The climber adjusts the body, sets the feet, and prepares for the next movement in order to reach a new hold. There, the two hands, $J_{7}$ and $J_{4}$, are fixed, and the moving feet seek their place on the lower grips. The video frames $F_{P}$ that make up this phase are identified as shown in Equation (11), following the rules set out in Figure 1.

(11) $$F_{P}=\left\{f_{i}\in C\;|\;f_{i}\in J_{M_{14}}\cup J_{M_{11}}\right\}$$

#### 4.6.2. Reaching

After preparation, the climber elongates the body as they reach for the next hold. While the feet, $J_{14}$ and $J_{11}$, remain stationary, they release one hand, $J_{7}$ or $J_{4}$, looking to grab the next hold in a vertical upward movement. Equation (12) defines the frame sequence for this phase.

(12) $$\begin{array}{c} F_{R}=\left\{f_{i}\in C\;|\;f_{i}\in J_{M_{8y}}\;\vee \;f_{i}\in J_{M_{7}}\cup J_{M_{4}}\;,\;f_{i}\in \bar{J_{M_{14}}}\cap \bar{J_{M_{11}}}\right\} \end{array}$$

#### 4.6.3. Stabilization

Once reaching is achieved, the hands remain static on the grips and now the body is lowered, so that this allows them to start a new climbing cycle. Frames belonging to this phase are identified when the hands are static, as shown in Equation (13).

(13) $$F_{S}=\left\{f_{i}\in C\;|\;f_{i}\in \bar{J_{M_{7}}}\cap \bar{J_{M_{4}}}\right\}$$

### 4.7. Error Detection

Error detection is carried out by considering the phase in which the climber is. For this study, we examined four common errors linked to the reaching phase, one linked to stabilization, and one to the preparation phase, which will be detailed in this section.

**Figure 6:**
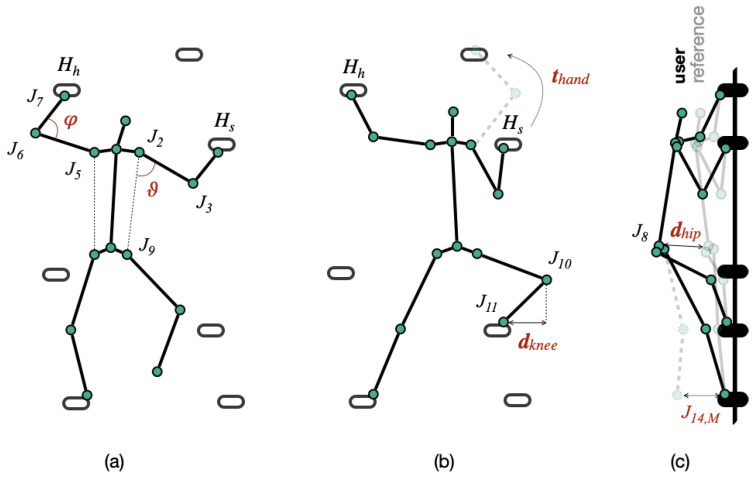
Angles and distances used in the error detection with holding hand ($H_{h}$) and supporting hand ($H_{s}$). (**a**) Measured angles for the elbow (φ) and shoulder (ϑ) in the decoupling and shoulder-relaxing errors, respectively. (**b**) Knee-to-ankle horizontal distance ($d_{knee}$) in the weight-shift error, and minimum time ($t_{hand}$) for the supporting hand in reaching-hand-supports error. (**c**) Hip-to-wall depth distance ($d_{hip}$) in relation to a reference climber for the hip-close-to-the-wall detection, and feet motion frames ($J_{M_{14,11}}$) for both-feet-set error.

Figure 6 shows an overview of the variables involved in our definition of the different errors. In addition to angles, distances, and time, the following two concepts are necessary before describing each error. The holding hand $H_{h}$ is defined as the hand that is in the higher position $p_{y}$ in relation to the other one. Complementarily, the supporting hand $H_{s}$ is the hand that is in the lower position $q_{y}$. These variables are defined in Equation (14) as follows:

(14) $$H_{h},H_{s}=\left\{\left(p,q\right):p,q\in J_{k\in \{7,4\}}\;,\;p_{y}>q_{y}\right\}$$

#### 4.7.1. Decoupling

The decoupling error is detected in the preparation phase $F_{P}$. There, the climber should not bend the arm of the holding hand to avoid loading the hand unfavorably, thus saving effort. The arm of the holding hand should be as straight as possible when the climber places the feet. For this evaluation, the elbow and shoulder angles, φ and ϑ, relative to the holding hand are constructed according to Equation (15).

(15) $$\varphi =\left\{\begin{array}{cc} ∡J_{7}J_{6}J_{5} & H_{h}=J_{7}\\ ∡J_{4}J_{3}J_{2} & H_{h}=J_{4} \end{array}\right.\;,\;\vartheta =\left\{\begin{array}{cc} ∡J_{6}J_{5}J_{12} & H_{h}=J_{7}\\ ∡J_{3}J_{2}J_{9} & H_{h}=J_{4} \end{array}\right.$$

The set of frames $E_{E}$ in which the angle φ or the angle ϑ are below the threshold $K_{\phi }$ or $K_{\theta }$, respectively, constitute the decoupling error detections, as shown Equation (16).

(16) $$E_{E}=\left\{f_{i}\in F_{P}\;|\;\varphi_{f_{i}}<K_{\phi }\;\vee \;\vartheta_{f_{i}}<K_{\theta }\right\}$$

#### 4.7.2. Reaching Hand Supports

While the climber is in the standing-up action, i.e., in the reaching phase $F_{R}$, the supporting hand $H_{S}$ should not be in motion for longer than $K_{t}$, thus stabilizing the body and using less energy. By evaluating the time $t_{hand}$ during which the supporting hand is in movement ${H_{S}}_{M}$, we assess the occurrence of the error as defined in Equation (17).

(17) $$E_{G}=\left\{f_{i}\in F_{R}\;|\;f_{i}\in {H_{S}}_{M}\;\wedge \;t_{hand}>K_{t}\right\}$$

#### 4.7.3. Weight Shift

The weight-shift error occurs when the climber stands up to grasp the next hold, but the hip does not move in the direction of the supporting hand $H_{s}$, so that the main body weight does not rest on the hold. The error is detected in the reaching phase $F_{R}$ by checking whether the knee does not pass in front of the supporting foot. To achieve this, we first determine the distance vector $d_{knee}$ between the knee and the supporting foot, the latter related to $H_{s}$ as given in Equation (18). Next, we identify the error by checking whether the *x*-component of $d_{knee}$ is less than a threshold $K_{d_{knee}}$, as expressed in Equation (19).

(18) $$d_{knee}=\left\{\begin{array}{cc} J_{14}-J_{13} & H_{s}=J_{7}\\ J_{10}-J_{11} & H_{s}=J_{4} \end{array}\right.$$

(19) $$E_{H}=\left\{f_{i}\in {F_{R}}_{k}\;|\;d_{{knee}_{x}}<K_{d_{knee}}\;,\;k=\left[\frac{1}{4},\frac{3}{4}\right]\#F_{R}\right\}$$

Note that the knee, by the nature of the leg’s movement, may not pass in front of the supporting foot at the beginning and end of the reaching phase; for this reason, we only consider the central frames of the $F_{R}$ sequence, i.e., the frames of the two central quartets.

#### 4.7.4. Both Feet Set

While standing up in the reaching phase $F_{R}$, both feet should be placed either on the wall or on the holds to stabilize the body. One leg should support the lateral displacement of the hips and the straightening, while the other leg is mainly stabilizing. In the validation, the set of frames where one of the feet is in motion or not located on the wall are marked with error, as shown in Equation (20).

(20) $$E_{P}=\left\{f_{i}\in F_{R}\;|\;f_{i}\in J_{14,M}\cup J_{11,M}\;\vee \;J_{14z,f_{i}}>0\;\vee \;J_{11z,f_{i}}>0\right\}$$

#### 4.7.5. Hip Close to the Wall

To assess this error type, that occurs in the reaching phase $F_{R}$, we need to compare the climber’s hips position in a recording $C^{\prime }$ with respect to another $C^{\prime \prime }$ that is selected as reference on the same climbing route. For this purpose, we rely on the alignment of the videos $W_{C}$ that provides us with the corresponding frames in both sequences to perform the comparison of the *z*-position of both climbers’ hips $J_{8}$. Hence, we can calculate the perpendicular distance of the hips ${J_{8}^{\prime }}_{z}$ and ${J_{8}^{\prime \prime }}_{z}$ to the climbing wall in each video, according to the frame tuples given by $W_{C}$ as indicated by Equation (8). If the difference between the distance to the wall of $J_{8}^{\prime }$ and $J_{8}^{\prime \prime }$ is greater than a threshold $K_{d_{hip}}$, the frame in $C^{\prime }$ is flagged with error, following the description in Equation (21).

(21) $$E_{W}=\left\{f_{i}\in F_{R}\;|\;J_{8,f_{j}}^{\prime \prime }-J_{8,f_{i}}^{\prime }⩾K_{d_{hip}}\;,\;\left(f_{i},f_{j}\right)\in W_{C}\right\}$$

#### 4.7.6. Shoulder Relaxing

After reaching, the climber enters the stabilization phase $F_{S}$ and the arm of the new holding hand should be stretched again. If this arm $H_{h}$ remains locked, i.e., the elbow and shoulder angles do not open, the so called shoulder-relaxing error will be observed. This error is established for the elbow and shoulder with respect to $H_{h}$ when their angles are below a certain threshold, $K_{\phi }$ and $K_{\theta }$, respectively, as shown in Equation (22).

(22) $$E_{N}=\left\{f_{i}\in F_{S}\;|\;\varphi_{f_{i}}<K_{\phi }\;\vee \;\vartheta_{f_{i}}<K_{\theta }\right\}$$

Note that Equations (16) and (22) share the calculation of the angles φ and ϑ, but differ in the phase where the error occurs.

### 4.8. Climbing Application for Users and Trainers

The proposed rules for identification of the six climbing errors defined in the previous section were coded in C++ within an application developed for the iPad Pro 4th Generation. This application allowed us to carry out the recording and processing of the climbing video sequences, starting with the recording of a reference route, which is performed by an experienced climber as a trainer. The reference recording demonstrates the proper pose to be adopted by the climber in each of the preparation, reaching, and stabilization phases, as well as the correct movements of hands, arms, waist, feet, and legs in each of the transitions between these three phases. Similarly, the application allows a user to select the reference video sequence and make multiple recordings of themselves in order to obtain feedback on their movements and hips position relative to the wall, when compared to the reference sequence.

To capture the video and process the images we use Apple’s ARKit framework. ARKit allows the recording of RGB videos with the possibility of mapping reticular sections of the image with the LiDAR sensor measurements. The sensor uses a global matrix of 576 points and, in conjunction with the integrated motion sensors, builds the depth map for each video image [38]. We choose an image resolution of 1440 × 1920 pixels, considering that ARKit does not provide the raw information of the depth measurements, but resorts to a combination of the image pixel color and the LiDAR information to build the depth map of the scene by means of an AI algorithm [39]. From this depth grid we developed the algorithm to create an ordered point cloud, whose density depends directly on the selected frame rate. The grid was established as a 118 × 158 points mesh, which resulted from the threshold that allowed us to process in parallel the maximum number of frames with an available memory of 4 GB.

Below, in Section 5.4, is an example of the graphical feedback that climbers receive on their recordings, indicating the errors made and the error count, allowing them to compare their performance on each new attempt.

## 5. Evaluation

The test setup is presented below, followed by the methodology applied in the evaluation, and concluding with an analysis of the obtained results.

### 5.1. Evaluation Setup

We selected four experienced climbers, defined as those who had mastered the six climbing techniques we were interested in evaluating. These individuals, of different sizes, were tested on three reference routes designed to demonstrate the three climbing phases and detect the six possible climbing errors described in this paper. The climbers were instructed to execute a climbing route three times: first time trying to perform a clean climb, i.e., free of errors; second time, having at least one error per phase or transition; and finally, executing a middle performance, with not a specific number of correct or wrong movements and poses. Overall, 21 RGB-D videos of an average duration of 20 s were recorded at 60 FPS, which were manually labeled by defining the range of frames where the climbing error was evident. This labeling included 58 decoupling, 32 weight-shift, 46 hip-close-to-the-wall, 16 both-feet-set, 35 shoulder-relaxing, and 48 reaching-hand-supports error actions.

### 5.2. Evaluation Methodology

**Figure 7:**
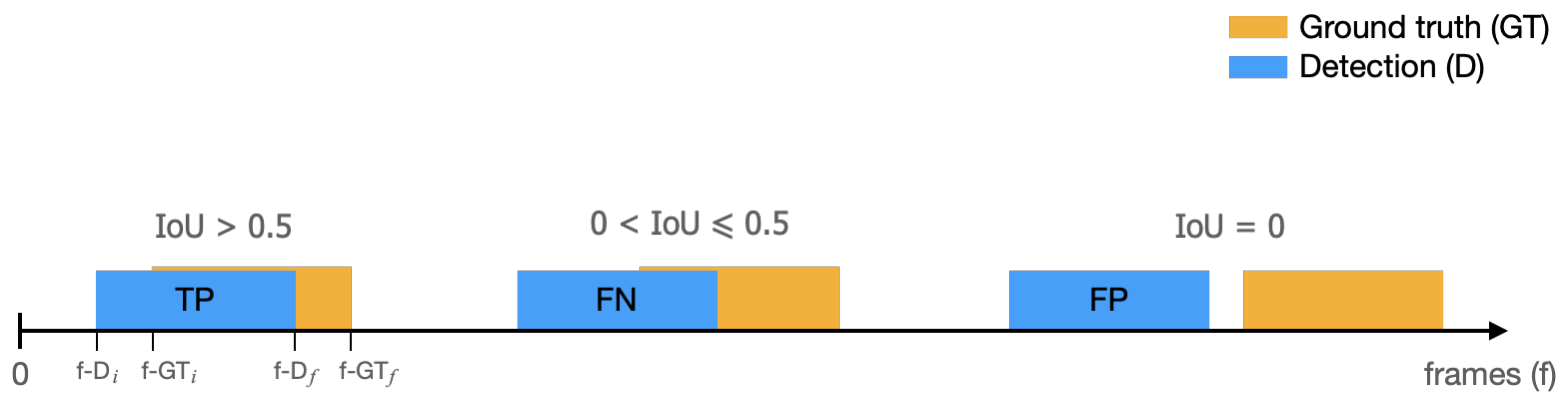
Schematic of the overlap between detection range frames and ground truth for a given climbing error. Here, IoU>0.5 is used as the threshold to distinguish TP from FN.

The six defined rules were applied in parallel on each video recording. For each frame of the video sequence, the climbing phase in which the climber was located was determined and the errors corresponding to that phase were evaluated. This procedure results in a set of frame-tuples for each kind of error, indicating the video sequence intervals in which the climbing error is detected. Afterwards, these detection frame-tuples were overlaid on the corresponding set of ground truth frame-tuples, as shown in Figure 7, to apply a decision index and, thus, obtain a set of TP, FN, and FP values used to construct the precision–recall curves, as explained below.

The error counting was performed per phase, i.e., if more than one error-tuple type is detected in a given climbing phase, this is counted as a single error for the phase and for such an error type.

#### 5.2.1. IoU Index

To assess the accuracy of our climbing error evaluations, we apply the Jaccard similarity index, also known as the intersection over union (IoU) method. As shown in [40], the IoU is a measurement commonly used in ML to evaluate object detectors on specific image datasets. The method compares the ground truth bounding box $B_{gt}$ of a target with the predicted bounding box $B_{p}$ by dividing the overlap between $B_{gt}$ and $B_{p}$ by their union, as shown in Equation (23). The IoU index measures how close the prediction bounding box is to the ground truth, with values ranging from 0 to 1 indicating the matching: 0 for no overlapping and 1 for a perfect match.

(23) $$IoU=\frac{|B_{gt}\cap B_{p}|}{|B_{gt}\cup B_{p}|}$$

In our experiments, we use the 1-D case of the IoU method, where the bounding boxes consist of the ground truth and detection frame ranges. The IoU index gives us a measure of the overlap between the labeled video frames for each climbing error and the detection range produced by our algorithm. To differentiate true positives (TPs) from false negatives (FNs) we use a variable threshold for IoU greater than zero; the variation of which will later allow us to find the optimal value, and we assume false positives (FPs) if IoU=0, as shown in Figure 7.

#### 5.2.2. Precision–Recall Curves

According to [41], in a rare event problem composed of unbalanced data, the receiver operating characteristic (ROC) score can be misleading, and the precision–recall curve (P-RC) is a better choice for assessing the model. We consider the climbing error detection an unbalanced data problem, as the percentage of non-events of a given error type is significantly higher than the percentage of error events; therefore, we have preferred the P-RC analysis to the ROC score.

To perform the P-RC analysis, we determined the number of TPs, FNs, and FPs for each type of climbing error in each of the 21 videos in the evaluation set. To obtain P-RC performance metric we used a series of 20 thresholds between 0 and 1 for the IoU index, and calculated the *precision* and *recall* indicators according to Equation (24).

(24) $$\begin{array}{c} precision=\frac{TP}{TP+FP}\;,\;recall=\frac{TP}{TP+FN} \end{array}$$

### 5.3. Results and Discussion

The conditions set out in Section 4.7 for the identification of the climbing errors include different thresholds, which are defined here as independent constants for each case. Table 1 presents the values for these constants, which were determined on the basis of the specifications given by our climbing expert and the fine-tuning carried out after many tests.

**Figure 8:**
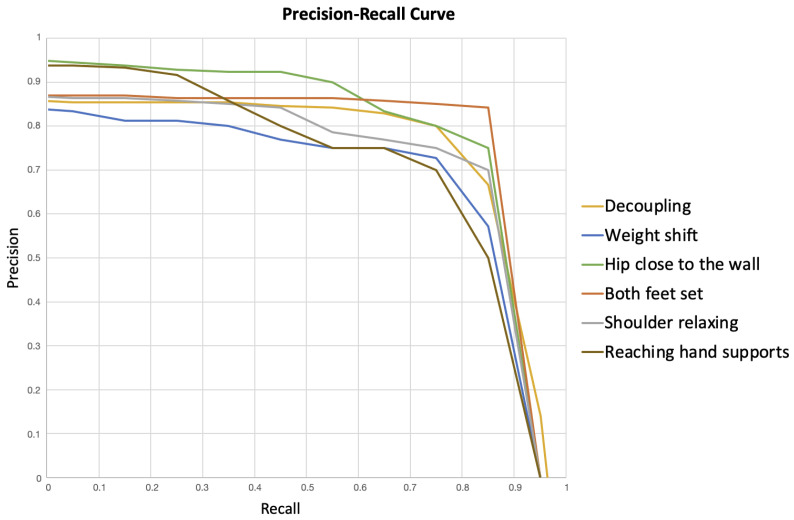
Precision–recall curves for the six error evaluations.

Once the experiments had been carried out, the data collected, and the P-RC analysis performed, as detailed in the previous sections, we found that the proposed algorithms generally produced reliable results with optimal points for precision and recall above 0.7 and 0.75, respectively, as shown in Figure 8.

The most accurate validation occurs for the both-feet-set error type, which has an optimal IoU threshold of 0.85, as shown in Table 2. This is consistent with the fact that the algorithm does not include any additional rules apart from the limb movement and position verification, which in turn means that the TP rate is directly related to a good detection of the climber’s pose where the lower extremities do not present major occlusions. On the other hand, the most complex validation turns out to be the weight-shift error, with an IoU of 0.4. The rules for this error type include the distance knee–ankle $d_{knee}$ evaluated in the middle of the reaching phase $F_{R}$, for which we apply the validation within the middle two quartets of the frame interval. The difficulty in this detection lies in the fact that the climber does not always move the hip laterally when the next target to be grasped is within arm’s reach in an upward direction, therefore, the expected condition is not fulfilled, and an FP is produced.

Decoupling and shoulder relaxing show similar results in correctly validating the predicted $K_{\epsilon }$ and $K_{\theta }$ angles for the elbow and shoulder, respectively. This can be understood by noting that both perform the measurements on the arms, which may be influenced by similar conditions such as light or jittering in the detection of their joints. Nevertheless, shoulder relaxing has lower precision due to the arm being more exposed to occlusions in the stabilization phase $F_{S}$.

The reaching-hand-supports scenario presents problems with high thresholds for the IoU index, and the results are more reliable when a low IoU is used to discern between TPs and FPs. This is explained by considering that the wrist joint is often hidden and, therefore, the estimated position varies within the time interval $K_{t}$, which induces motion detections that trigger error marking and increase the FP rate.

The hip-close-to-the-wall error is a direct result of the DTW algorithm and the error mark depends on the $K_{d_{hip}}$ constant. The latter measures the distance of the climber from the climbing wall according to the reference, but as some test subjects had larger body proportions than the reference climber, the $K_{d_{hip}}$ value cannot be applied consistently for all climbers. Nevertheless, the overall result is accurate, with an optimal IoU of 0.9. In addition, video synchronization with hip tracking, which relies on the same DTW algorithm, occurs correctly.

Finally, the results show that the detection of the different types of climbing errors is accurate in a wide range of cases, although FPs are still relevant, they occur mostly in those parts of the video where the detection of hands or feet shows fluctuations, due to jittering or occlusions. However, measurements on joints other than these limbs, such as the CoM, are reliable, with a low rate of FPs; this is demonstrated by the effectiveness of using DTW to synchronize two video sequences and allow comparison of climbers in the same position.

### 5.4. Feedback Results for Climbers

**Figure 9:**
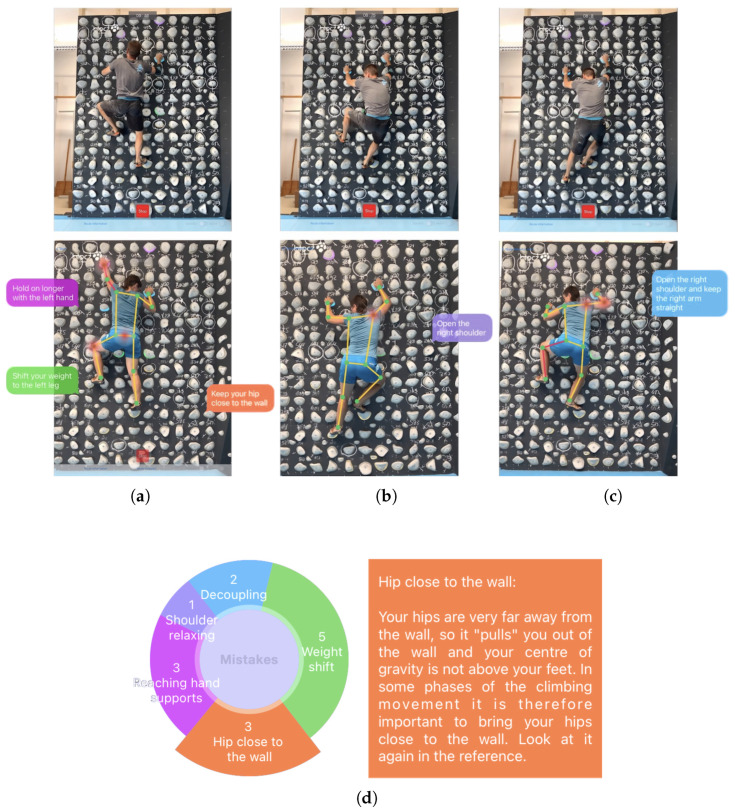
Application feedback for novice climbers. Different errors are presented per climbing phase, and a summary of the total errors with hints to improve the next attempt. Image source [29]. (**a**) Errors in reaching phase; (**b**) error in stabilization phase; (**c**) error in preparation phase; (**d**) climbing error summary.

The developed application provides climbers with graphical feedback on the errors made in the three climbing phases. As the climber reviews the video, they can see the synchronization of their movements with those of the selected trainer on the reference route. This is specially helpful for novice climbers, who can compare their moves at each route step, moving the video forward and backward at will. In the video reviewing, the climber goes through the different climbing phases and is accompanied by feedback messages on the wrong movements performed, if any. These messages tell the climber how the trainer expects the movement to be executed, and the errors are counted to present a summary of the total number of errors made by type, as shown in Figure 9. This summary also includes a description of each error type, useful for understanding how each was determined.

## 6. Conclusions and Future Work

Electronic devices that are affordable for a large part of the population increasingly allow us to develop innovative applications that use non-invasive sensors, as is the case with the iPad Pro with LiDAR sensor. Thanks to this availability, we have been able to implement ideas that contribute to the development and popularization of sporting trends such as sport climbing and its variant bouldering. In this work, we have presented not only an application of sport theory that allows the user to improve themselves by following basic concepts in climbing, but we also propose rules for defining transitions between climbing phases based on the position and movement of the body extremities.

The movement and position evaluation of the various body joints is made possible by the continuous development of HPE frameworks. However, the jittering and occlusion of the body parts play an important role in obtaining an accurate set of measurements. Therefore, in the proposed solution we have made pose detection independent of the used device to enable continuous improvement of our application by being able to integrate new HPE algorithms as new techniques become available.

An essential aspect of our proposal, both in the climbing error detection algorithms and in the application design, is to make our HPE module invariant to the scenario in which it is used. We achieve this using 3-D modeling of the climber and the climbing wall, which provides a mechanism for making recordings with different camera configurations from different viewpoints. However, we still depend on the sensitivity of the sensor, in this case LiDAR, which gives us its best resolution within the range of 4 to 6 m from the target. This limits the application to some extent when the measurement is carried out statically, but proposes a new requirement based on a moving sensor. A future scenario would be the application of our proposed methods in the sport climbing discipline named speed climbing, in which the climbing wall dimensions are much larger and would require, if not several cameras, then a moving camera following the climber during the ascent.

The movement modeling of the body joints as 3-D signals allowed us to apply techniques known in the field such as DTW and standard score, and also simplified the number of variables by considering the climber as a set of 19 joints. This also allowed us to graphically and statistically compare different situations of the climbers’ movements when making transitions between climbing phases, thus simplifying the validations and the number of variables used there. For a next iteration of this work, we will extend the developed rules by replacing the used constant thresholds with models based on the proportions and constraints of the human body.

To conclude, the perspectives regarding the development of useful applications for athletes are very wide, in particular for sport climbing. In a next project, the present work will serve as a basis for training a predictive model that not only highlights wrong climbing actions, but also suggests to the user the next move, considering the rules, to obtain the best and most energy-efficient pose.

## Figures and Tables

**Table 1:** Values of the thresholds used in the defined validations.

Validation	Constant	Description
Decoupling and	$K_{\epsilon }=130^{\circ }$	Threshold angle for open elbow
shoulder relaxing	$K_{\theta }=120^{\circ }$	Threshold angle for open shoulder
Reaching hand supports	$K_{t}=1\;s$	Threshold grip time of the supporting hand
Weight shift	$K_{d_{knee}}=20\;\mathrm{cm}$	Threshold distance from knee to foot
Hip close to the wall	$K_{d_{hip}}=5\;\mathrm{cm}$	Threshold hip distance to the reference climber’s hip
Motion segmentation	$K_{\mu }=30$	Threshold number of frames for a valid movement

**Table 2:** Optimal thresholds for the IoU index differentiated by climbing error type.

Climbing Error	Optimal IoU	Optimal Precision	Optimal Recall
Decoupling	0.8	0.8	0.75
Shoulder-relaxing	0.75	0.7	0.85
Reaching-hand-supports	0.45	0.7	0.75
Weight-shift	0.4	0.73	0.75
Hip-close-to-the-wall	0.9	0.75	0.85
Both-feet-set	0.85	0.84	0.85

## Data Availability

Not applicable.
